# Vitro culture of axe-head glochidia in pink heelsplitter *Potamilus alatus* and mechanism of its high host specialists

**DOI:** 10.1371/journal.pone.0192292

**Published:** 2018-02-15

**Authors:** Hai Bo Wen, Wu Jin, Xue Yan Ma, Bing Qing Zheng, Pao Xu, Liang Xu, Dan Hua, Xin Hua Yuan, Ruo Bo Gu

**Affiliations:** 1 Wuxi Fishery College, Nanjing Agriculture University, Wuxi, Jiangsu, China; 2 Key Laboratory of Genetic Breeding and Aquaculture Biology of Freshwater Fishes-Ministry of Agriculture, Freshwater Fisheries Research Center, Chinese Academy of Fishery Sciences, Wuxi, Jiangsu, China; 3 Sino-US Cooperative International Laboratory for Germplasm Conservation and Utilization of Freshwater Mollusks, Freshwater Fisheries Research Center, Chinese Academy of Fishery Sciences, Wuxi, Jiangsu, China; 4 Cumberland River Aquatic Center, Tennessee Wildlife Resources Agency, Nashville, Tennessee, United States of America; Shanghai Ocean University, CHINA

## Abstract

The basal media M199 or MEM was utilized in the classical method of vitro culture of glochidia where 1–5% CO_2_ was required to maintain stable physiological pH for completion of non-parasitic metamorphosis. The classical method encounters a great challenge to those glochidia which undergo development of visceral tissue but significantly increase in size during metamorphosis. The improved in vitro culture techniques and classical methods were firstly compared for non-parasitic metamorphosis and development of glochidia in pink heelsplitter. Based on the improved method, the optimal vitro culture media was further selected from 14 plasmas or sera, realizing the non-parasitic metamorphosis of axe-head glochidia for the first time. The results showed that addition of different plasma (serum) had significant effect on glochidial metamorphosis in pink heelsplitter. Only glochidia in the skewband grunt and red drum groups could complete metamorphosis, the metamorphosis rate in skewband grunt was 93.3±3.1% at 24±0.5°C, significantly higher than in marine and desalinated red drum. Heat-inactivated treatment on the plasma of yellow catfish and *Barbus capito* had significant effect on glochidia survival and shell growth. The metamorphosis rate also varied among different gravid period, and generally decreased with gravid time. Further comparison of free amino acid and fatty acid indicated that the taurine of high concentration was the only amino acid that might promote the rapid growth of glochidial shell, and the lack of adequate DPA and DHA might be an important reason leading to the abnormal foot and visceral development. Combined with our results of artificial selection of host fish, we tentatively established the mechanism of its host specialists in pink heelsplitter for the first time. This is the first report on non-parasite metamorphosis of axe-head glochidia based on our improved vitro culture method, which should provide important reference to fundamental theory research of glochidia metamorphosis and also benefit for better understand of mechanism of host specialists and generalists of Unionidae species.

## Introduction

Most Unionidae species are obligated to undergo short period parasite on special fish or amphibian to complete the metamorphosis and maintain nature reproduction [1–3]. Since Isom & Hudson established the glochidial vitro culture method [4], it was much improved on preparation of glochicdia, usage of antibiotics and plasma, change of media, dilution technique and so on [5–8], and formed the classical method for glochidial vitro culture. Compared traditional fish infestation, the vitro culture has many advantages such as large numbers of individuals produced, lower cost per juvenile, efficient use of glochidia, low failure rate of propagation ect., and has improtant application value especially for those species which is lack of host fish information [9]. It will benefit to artificial propagation and conservation of Unionida in the world, especially for endangered species without information of host fish, and provid an important technical method for fundamental research on its metamorphosis.

The basal media M199 or MEM was generally utilized in the classical method of vitro culture of glochidia and 1–5% CO_2_ was required to maintain stable physiological pH for completion of its non-parasitic metamorphosis [7]. There are three types of glochidia including hooked, hookless and axe-head accroding to Howells et al [10]. At present, 41 Unionidae species had successful report on non-parasitic metamorphosis through the classical virto culture method [9]. All these species tested possess large valves as glochidia belonging to hooked or hookless type, and only undergo development of visceral tissue with little growth in size. Based on the classical vitro culture technique and theroy, it is a great challenge to complete metamorphosis of those glochidia which significantly increase in size during metamorphosis. For example, the genus *Leptodea* possess glochidia less than 60 μm in shell length that must develop not only visceral tissue, but amass an increase in shell length four-fold before they become fully developed juveniles [7]. Therefore, it is a hotspot and difficulty in the study of glochidia vitro culture. On the other hand, Isom & Hudson [11]showed that the fish species of plasma used had little effect on the development of the glochidia, whereas they had significant difference on metamorphosis rate in some Unionidae species [6,7,12]. All results indicated that the nutritional requirement greatly varied among different species during metamorphosis development. Based on the results of Owen [7], apart from quality of glochidia and control of bacteria and fuga contamination, the selection of plasma or critical nutrition should become an improtant sally port to the metamorphosis of those glochidia.

The pink heelsplitter, *Potamilus alatus*, is a large native freshwater pearl mussel in North America, belonging to the Lampsilinae subfamily. Nature atropurpureus pearls were found in its original habitats [13], and our artificial pearl operation experiment have showed that the pink heelsplitter was an excellent freshwater mussel species for high quality atropurpureus pearl production [14]. For development of atropurpureus pearl culture in China, Freshwater Fisheries Research Center (FFRC) introduced in 2012 hundreds of juvenile pink heelsplitter to carry out artificial domestication [15]. It was proved the pink heelsplitter had the highest host specificity, and freshwater drum *Aplodinotus grunniens* was reported as the sole host fish [16–19]. The absence of freshwater drum in China seriously restricts the large scale artificial breeding and the industrialization of atropurpureus pearl production. Recently, we selected out the red drum as potential host fish for pink heelsplitter from 16 fish species, but the infested red drum showed high mortality rate [20]. Therefore, the vitro culture is a preferred method to achieve metamorphosis of pink heelsplitter. The glochidia of pink heelsplitter is axe-head, and two valves can’t completely shut down. The shell size and inner tissues of glochidia should grow during metamorphosis with long term duration [18], which is very similar to the genus *Leptodea*. Therefore, it is very difficult to achieve vitro culture of pink heelsplitter.

The basal media L15 was firstly utilized in vitro culture of hookless glochidia in triangle pearl mussel *Hyriopsis cumingii* [21], and also obtained excellent results in cockscomb mussel *Cristaria plicata* with hooked glochidia [22]. In present study, the improved vitro culture techniques and classical methods were compared for non-parasitic metamorphosis and development of glochidia in pink heelsplitter. Further, effects of 11 fish species of plasma and 3 commercial animal sera, proportions of two plasma mixture, reserved time, heat-inactivation treatment and gravid period on its metamorphosis were investigated respectively. The variation of morphology of glochidia also was observed in the process of in vitro culture. The nutrient component of four respective fish plasmas was analyzed and the critical nutrient limiting factors were also discussed in this paper. This brought first insight on non-parasite metamorphosis of axe-head glochidia through our improved vitro culture method, and made a breakthrough on the critical technique of artificial propagation of pink heelsplitter. Further combined with our results of artificial selection of host fish, we tentatively established the mechanism of its host specialists in pink heelsplitter for the first time. It should provide reference to fundamental theory research of glochidia metamorphosis and improve the in vitro culture technique, also have important value on the artificial propagation and conservation of Unionidae species of the world.

## Materials and methods

### Ethics statement

This study was approved by the Animal Care and Use Committee of Nanjing Agricultural University (Nanjing, China). The handling of mussels was conducted in accordance with the Guide for the Care and Use of Experimental Animals of China.

### Source of parent mussel and glochidia preparation

The juvenile pink heelsplitter with 5–14 mm in shell length was introduced from USA in 2012 and reached mature after two years cultivation. From mid-October in 2014, the marsupia of gravid female mussels were examined to identify mature glochidia according to previous study [18]. The basic information of gravid mussels was shown in Table 1. All glochidia were collected according to Hua [18], and washed for 3-5times in the culture media with L15 basal media and mixed antibiotics in the ratio of 4 to 1 according to the protocol of Owen [7].

**Table 1:** Metamorphosis rate of glochidia in vitro culture from different breeding stages.

Gravid mussel	Collection date	Total number of glochidia	Metamorphosis rate (%)	
			Range	Mean ± SD
N1	2014-11-21	25800	89.6∼95.8	92.9 ± 3.1^a^
N2	2015-3-4	29800	46.3∼58.5	50.8 ± 6.7^b^
N3	2015-4-10	19867	30.2∼37.8	34.8 ± 4.1^c^
N4	2015-5-1	2500	70.5∼72.5	71.2 ± 1.1^d^
N5	2015-5-1	23533	30.0∼34.2	32.8 ± 2.4^c^
N6	2015-5-1	37400	28.5∼37.1	34.2 ± 5.0^c^
N7	2016-3-17	31400	29.6∼34.8	31.6 ± 2.8^c^

Metamorphosis rates labeled with different letters are significantly different (*P*<0.05).

### Plasma preparation

Total of 11 fish were selected for plasma collection, including 7 kinds of freshwater fish, 2 kinds of marine fish and euryhaline fish respectively, and quarantined and fed daily for 2 weeks to ensure their health prior to the experiment. The basic information was shown in S1 Table. Fish blood was collected from the caudal vein in the tail area, using asyringe needle no.18 according to Ma *et al* [22]. All collected blood was pooled and centrifuged at 3000 r/min for 10 min, and the supernatant was passed through sterile filters (0.22μm pore size) twice. The plasma was stored at -80°Cfor follow-up experiments. The sera of bovine, equine and rabbit were produced by Sangon Co., Ltd., Shanghai, China.

### Comparison of two kinds of culture method

The difference of glochidial metamorphosis was compared by the improved and classical vitro culture method based on commercial medium L15 and M199 respectively. The culture formulas were shown in Table 2. The antibiotic solution mixture contained carbenicillin (100μg/ml), gentamycin sulfate (100μg/ml), rifampin (100μg/ml), and amphotericin B (5μg/ml) according to Isom and Hudson [4]. The glochidia(300±10/dish) were cultured in a sterile culture dish (15×60 mm)at 24±0.5°C, and the medium was refreshed every 3 days. Three experimental replicates were conducted for each group. After 20days culture, the aerated water was added to the culture dish to wake up the transformed juvenile mussel, and then the survival and metamorphosis rate were determined according the formula indicated below and described by Uthaiwan *et al* [6].

Thesurvivalrate=thenumberofsurvivalglochidia/totalnumberofglochidia×100% (1)

Themetamorphosisrate=thenumberoftransformedjuvenile/totalnumberofglochidia×100% (2)

**Table 2:** Improved and classical method for vitro culture of glochidia.

Protocol	Base media		Culture Condition		Antibiotic Cocktail (ml)
	Name	Volume(ml)	Instrument	CO_2_Supply	
Classical	M199	2	CO_2_ Incubator	5%	0.5
Improved	L-15	2	Temperature Control Incubator	no	0.5

### Effect of plasma addition on glochidial metamorphosis

#### Mono plasma addition

Each artificial medium contained the basic medium L15, the mono plasma or serum and antibiotics/antimycotics at the ratio of 2: 1: 0.5, and the total volume in each dish was 3.5ml. There were 11 kinds of fish plasmas, bovine, equine and rabbit sera utilized to select the optimal formula for metamorphosis of pink heelsplitter, and the other culture conditions were described as above improved method. The survival and metamorphosis rate were calculated after 20 days culture. All glochidia for vitro culture were collected from N1 gravid mussel (show in Table 1).

#### Mixture of two kinds of fish plasma

Based on the above results, the plasmas of skewband grunt and common carp were selected and mixed at 4 different ratios (show in Table 3). The vitro culture method and formula were described as above improved method, and three experimental replicates were conducted for each group. The glochidial metamorphosis was calculated after 20 days culture. All glochidia for vitro culture were collected from N2 gravid mussel (show in Table 1).

**Table 3:** Comparison of metamorphosis of glochidia of pink heelsplitter in vitro culture in addition of two mixed fish plasmas in the end of culture time.

Conditions	Fish plasma (ml)		Percentage of transformation(%)	Description of transformation			
	*H*.*nitens*	*C*. *carpio*		Shell growth	Foot development	Creeping	Activity
A	1	0	51.0±5.1^a^	Normal	Normal	Y	Best
B	0.8	0.2	5.6±1.4^b^	Fast	Normal	Y	Good
C	0.5	0.5	3.5±1.0 ^b^	Faster	Normal	Y	Bad
D	0	1	0	Fastest	Abnormal	N	No

Percentage of transformations labeled with different letters are significantly different (*P*<0.05).

#### Reserving time of fish plasma

Based on the above experimental results, the skewband grunt plasma reserved for 11months and newly prepared in one month were utilized to test the difference of glochidial metamorphosis. The vitro culture method and condition were also described as above improved method. The glochidial metamorphosis and abnormal rate were determined according to the formula indicated below after 20 days culture. All glochidia for vitro culture were collected from N7 gravid mussel (show in Table 1).

Theabnormalrate=thenumberofabnormaljuvenile/thetotalnumberofglochidia×100% (3)

#### Heat-inactivated treatment on fish plasma

According to the difference of glochidial survival in 14 plasmas, we selected the plasmas of yellow catfish and *B*. *capito* for heat-inactivated treatment. Each plasma sample was divided into two same groups, one group was heat-inactivated in a 56°Cwater bath for 30 min, and another group as control. The vitro culture method and condition were also described as above improved method. The difference of survival and metamorphosis of glochidia were observed and compared between heat-inactivated and control group. All glochidia were collected from N6 gravid mussel (show in Table 1).

### Differences in glochidial metamorphosis at different breeding stages

There were 1–3 gravid mussels randomly selected for glochidia collection at the different breeding stages (show in Table 1). The vitro culture method and condition were also described as above improved method, and the newly prepared plasma of skewband grunt was used. All glochidia of each group were collected from only one gravid mussel, and three experimental replicates were conducted for each group. The total number of glochidia in each gravid mussel and metamorphosis rate were calculated.

### Morphological observation of glochidia and transformed juvenile

Small samples of glochidia were collected from the dish for morphological comparison in the different vitro culture stages respectively. The optical microscope (Olympus CX41) and scan electronic microscope (Hitachi 3000N) were employed for observation of glochidial microstructure. The procedure of sample preparation for SEM was consistent to Wen *et al* [15].

### Determination of pH and osmotic concentration in different culture medium

Based on the differences of glochidial metamorphosis, the pH and osmotic concentration of 9 representative culture formulas were measured. EcoTestr pH2 (EUTECH)and OSMOMAT 030 (Gonotec) were employed respectively.

### Determination of biochemical components in representative fish plasmas

There were 5 kinds of fish species selected for determination of biochemical components based on the characteristics of glochidial metamorphosis, including skewband grunt, common carp, tilapia, marine and desalinated red drum. The filtered plasma from 10 individuals of each fish species was examined. The contents of K^+^, Na^+^, Cl^-^, Ca^2+^, P, Mg^2+^, TP, TG, TCh, Glu, LDL and HDL were determined according to Ma *et al* [21]. High Performance Liquid Chromatography (Aglient1100) was used to determine free ammonia acid according to the national standard of JY/T019-1996 and GB/T5009.124–2003. The standard of C11:0 was added into the dried plasma sample as internal standard. Subsequently, the sample was shock and extracted for 3 times, then filtrated and dried before inspection. GC-2010 gas chromatography (Shimadzu) was employed to determine the composition of fatty acid according to GC/FID method. Three replicates were conducted for each of fish plasma.

## Results

### Effects of vitro culture method on glochidial metamorphosis

**Fig 1:**
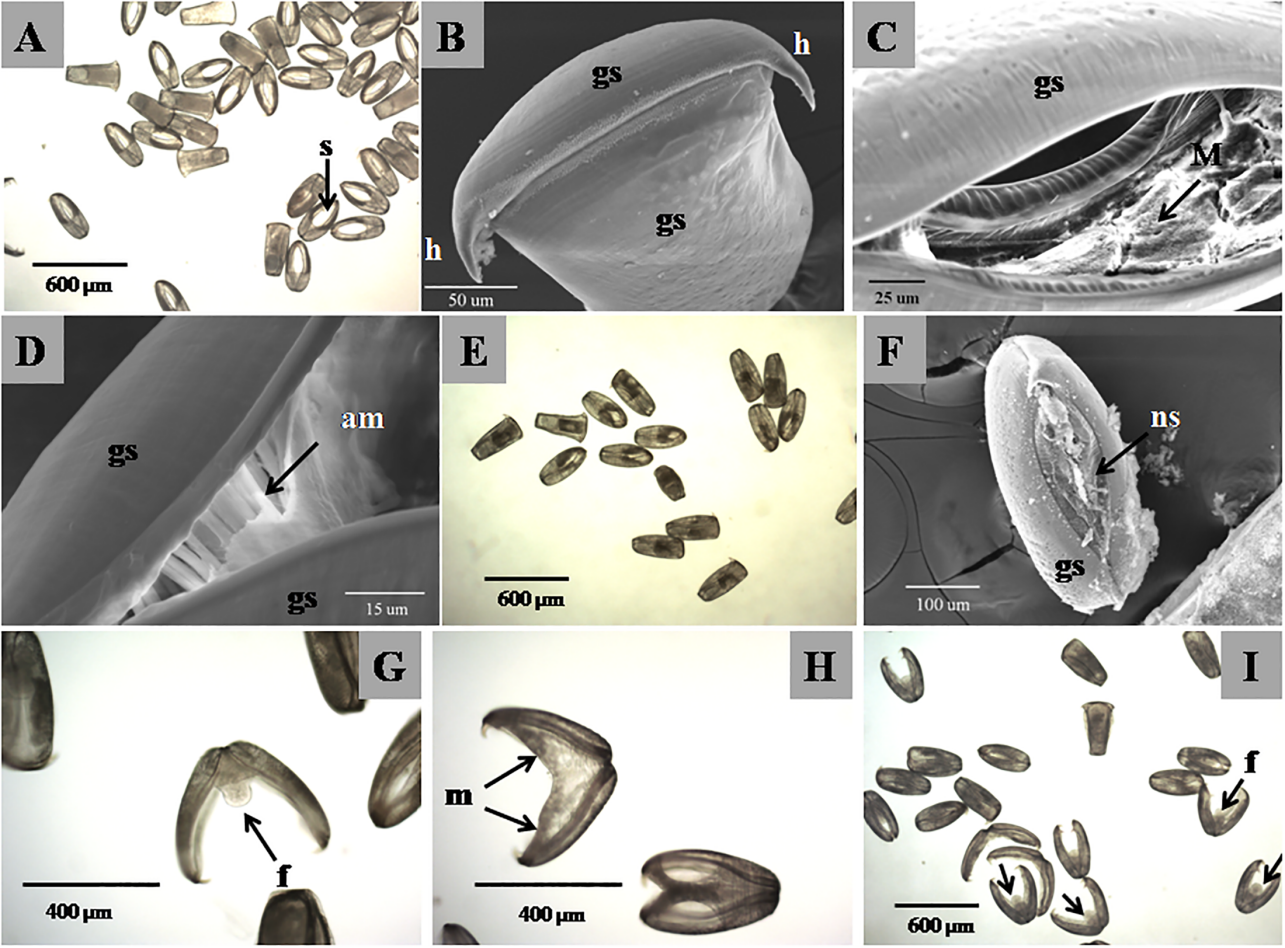
Comparison of metamorphosis of glochidia of pink heelsplitter in two different vitro culture methods. ns, new shell; gs, glochidial shell; s, space; f, foot; h, hook; m, mantle; am, adductor muscle.

All glochidia remained closed in the classical and improved culture method at the beginning (Fig 1A). The glochidia of pink heelsplitter had axe-head with relative narrow dorsal and wider ventral, and there were two lanciform hooks located at the lateral margins of the ventral flange of each shell. The double shells had little difference in the size which can be identified from ventral margin of closed glochidium (Fig 1B), and can’t completely close due to its curvature (Fig 1C), so that the internal tissues of glochidia were kept in circulation with the culture medium. There were amount of adductor muscle fiber bundles located in the near umbo (Fig 1D), which implement the movement of shell closing. In the first day, there was no significant difference in two vitro culture methods at 24±0.5°C and found that internal tissue and anterior and posterior edge of glochidia gradually grew and healed (Fig 1E). In sixth day, there was about 50%glochidial shell opened with different angle but not died in L15 group, while all glochidial shell remained closed with significant growth of the new shell in M199 group (Fig 1F), but the mantle tissue of some glochidia abnormally proliferated and inflated. To 12th day, there were still about 50%glochidial shell opened with short growing foot in L15 group (Fig 1G), while 80% glochidial mantle tissue abnormally expanded without foot and visceral mass developing in M199 group (Fig 1H). To 15th day, all glochidia were died in M199 group with rotting mantle, whereas 100% glochidia still survived but had undeveloped foot and visceral mass in L15 group (Fig 1I), and there was no significant morphological variation and normally transformed juvenile observed until to 20th day.

### Effects of different plasmas and sera on glochidial metamorphosis

**Fig 2:**
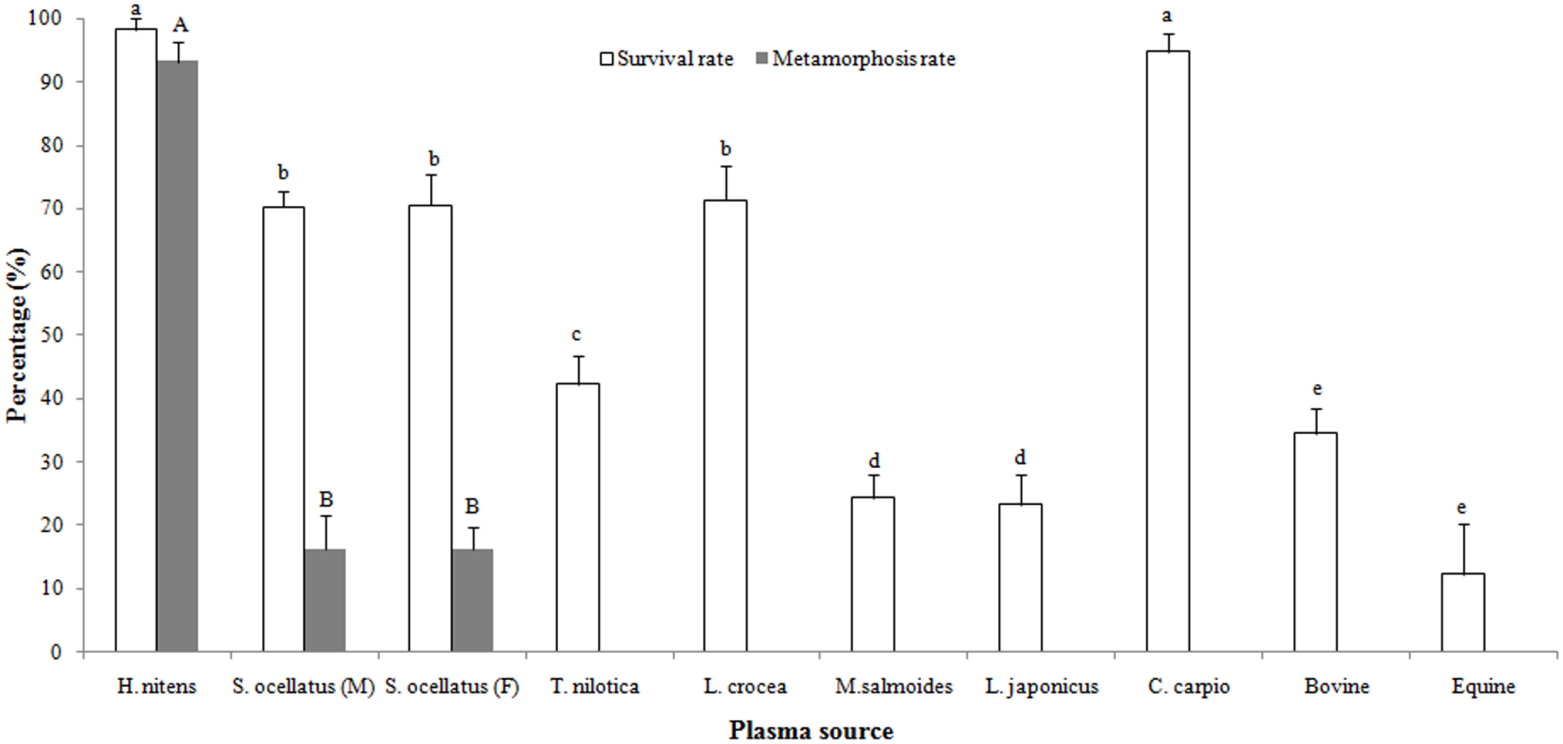
Percentage of survival and transformation of glochidia of pink heelsplitter in vitro culture with addition of 9 kinds of plasma respectively.

The results indicated that all glochidia died in mixed media with addition of plasma of yellow catfish, largemouth bass, *Barbus capito*, hybrid sturgeon and rabbit serum, whereas the survival rate was ranging from 12.4% to 98.3% in the other 9 groups, and completed metamorphosis only in skewband grunt and red drum (Fig 2). One factor ANOVA showed that the glochidial metamorphosis rate in skewband grunt group was 93.3+3.1%, significantly higher than marine and desalinated red drum (*P*<0.01), while there was no transformed juvenile mussel found in the other 11 groups. There was no significant difference between marine and desalinated red drum (*P* = 0.983).

**Fig 3:**
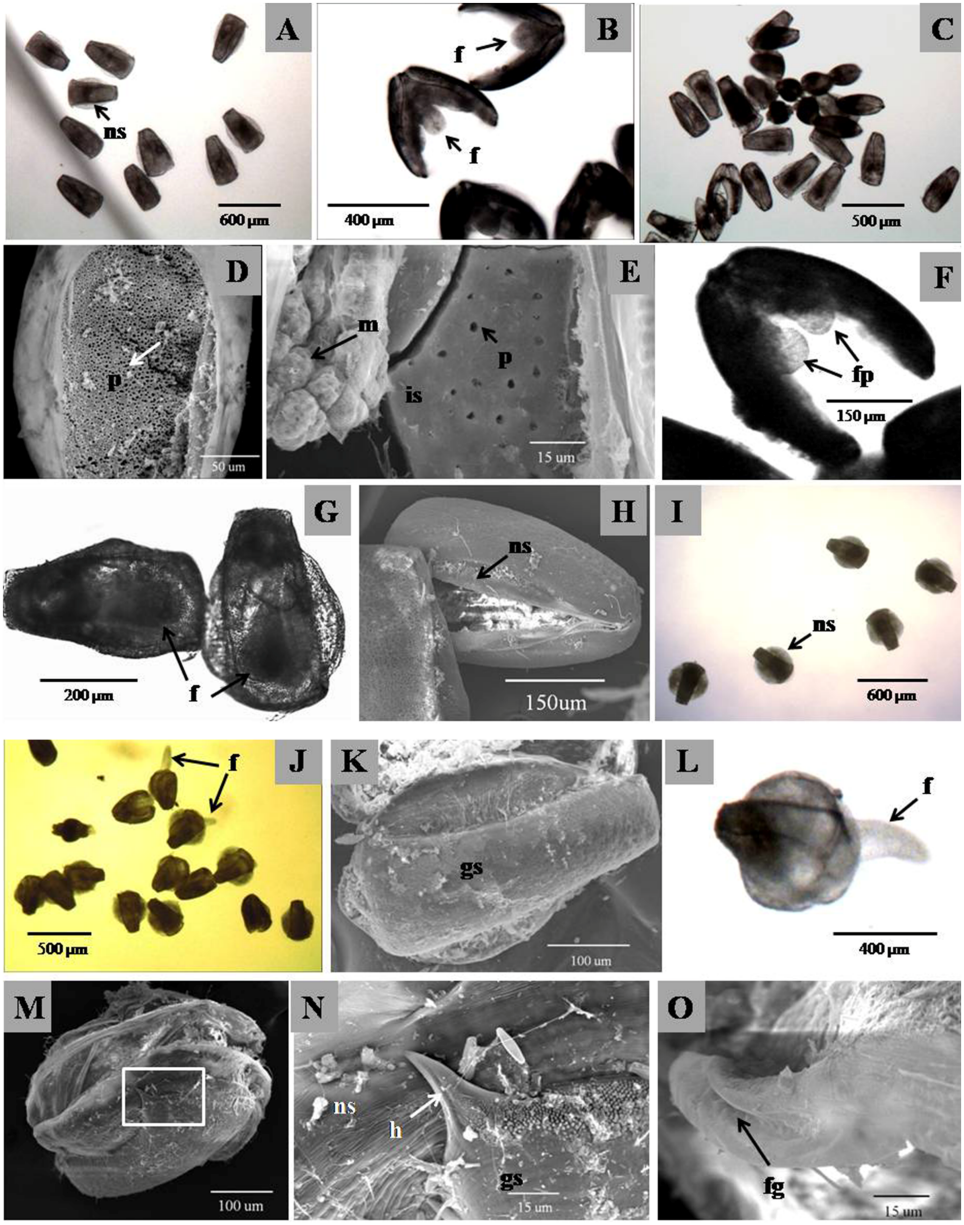
Morphological variation of glochidial metamorphosis in vitro culture with skewband grunt plasma. ns, new shell; gs, glochidial shell; is, interior of surface of shell; f, foot; fp, foot prototype; fg, foot groove; h, hook; m, mushroom cell; p, pit.

Total 14 kinds of plasma (serum) could be divided into 5 categories according to the difference of glochidial external morphology and internal tissue development. The first category included yellow catfish, *B*. *capito*, largemouth bass and hybrid sturgeon, and showed internal tissue of glochidia rapidly decayed and died without shell growth in the first two days. The second category included bighead carp, tilapia, yellow croaker and marine bass, where there was observed growth on external shell closed in the end of vitro culture with high survival rate but no complete metamorphosis. The third category included bovine, equine and rabbit, where the double shells of glochidia always kept opening and closing in the whole process of culture. In the early period, the glochidial shell in bovine and equine group had significant growth but with higher internal transparency comparing to skewband grunt (Fig 3A). To 7th day, all glochidia in equine and rabbit group had rotting internal tissue and died gradually, while the glochidia in bovine had undeveloped foot and visceral mass similar to that of control group without serum (Fig 3B). The fourth category included skewband grunt and red drum, where part of glochidia completed metamorphosis and freely crawled. In this category, the glochidial mantle cell expanded and became thicken, and the gap between the double shells healed in the first two days. The anterior and posterior of the double shells increased obviously from the third day, and some individuals connected together from the fourth day (Fig 3C). There were lots of fine hollow located on the inner shell (Fig 3D), while the number and depth of hollow significantly decreased in the survival glochidial inner shell where mushroom cells with uneven free surface were closely covering (Fig 3E). To 7th day, two foot paddles were observed and developed from the umbo position (Fig 3F) and able to move weakly, healed gradually and formed foot prototype in 9th day (Fig 3G). The new shell became nearly round, and the shell length of transformed juvenile increased to 2–3 times as long as glochidia after 20 days vitro culture (Fig 3H–3K). The juvenile mussel waked up in one day when transferred from the culture medium to freshwater, and crawled freely by foot (Fig 3J). There were lots of cilia observed on the foot surface and sparse cilia found in the edge of mantle tissues, but the gill filament had not yet developed completely. After one week cultivation, the shell of juvenile mussel also showed obvious growth from the ventral margin (Fig 3M), and two pairs of hook and papillae in ventral margin remained covering on the surface of new shell (Fig 3N). There was obvious foot groove found at the foot ventral margin (Fig 3O), and more cilia found along the edge the mantle which became thicken. The cilia of foot and mantle swung rapidly, and the crystalline style rotated uniformly. The fifth category was common carp, in which the glochidia had the fastest shell growth comparing to the normally transformed glochidia in fourth category, but showed more transparent shell, abnormal and expanded foot which only could stick out from the anterior and posterior side, hardly open and crawl.

### Effect of two fish plasma mixture on glochidial metamorphosis

One factor ANOV indicated that the metamorphosis rate in Group A was significantly higher than Group B with different proportion of common carp plasma (*P*< 0.05), but there was no significant difference between Group B and C (*P* = 0.367) (shown in Table 3). The metamorphosis rate in Group D was 0, whereas these untransformed glochidia had expanded and abnormal foot, but the fastest growth and largest shell length comparing to the other 3 groups. The transformed juvenile mussel in Group A had normal foot which can open and close, and crawled actively, and the juvenile of Group B had higher activity and lower abnormal rate than Group C.

### Effect of reserved time of skewband grunt plasma on glochidial metamorphosis

The metamorphosis rate in reserved plasma for 11months was 27.2+4.9%, was lower than newly prepared plasma (31.6+2.8%), but there was no significant difference (*P* = 0.25). There were some individuals of all un-transferred glochidia showed abnormal and expanded foot which was similar to the morphological characteristic in Group D. The abnormal rate of juvenile in the former group was 6.5±1.0%, significantly higher than the latter (1.2±1.1%) (*P* = 0.003).

### Effects of heat-inactivated plasma on glochidia survival

There were observed difference in glochidial survival and shell growth between heat-inactivated and control group of yellow catfish or *B*. *capito*. There was 30.0±1.4% glochidia found died with rotting internal tissue in all control groups in 2h, and all glochidia were died after 24h. However, all glochidia survived with the increasing double shells in all heat-inactivated groups even in 24h. To 10^th^ day, there were still 82.0±5.1% individuals survived with significant growing but ajar double shells in heat-inactivated yellow catfish plasma, and 55.0±4.5% survived in heat-inactivated *B*. *capito* plasma. Finally, there was no any transformed glochidium found in all groups after 20 days.

### Differences in glochidial metamorphosis at different breeding stages

Under the same vitro culture condition, the metamorphosis rate of glochidia collected in the early breeding stage (Nov) was significantly higher than the latter stages (from Mar to May) (*P* < 0.01), while there was no significance among the latter stages (*P*< 0.05)(shown in Table 1). However, there was observed difference in the metamorphosis rate of glochidia from different gravid mussel even collected at the same time. The metamorphosis rate from N4 gravid mussel was significantly higher than N5 and N6 (*P*< 0.01), and the number of glochidia incubated in the marsupium of N4 was about 10% of N5 and N6 (shown in Table 2).

### Difference of pH and osmotic concentration in different formulas

The results of pH and osmotic concentration of different formulas were shown in Table 4. The pH value of Group M0 was weak alkaline, higher than Group L0, While the osmotic concentration of the former was significantly lower than the latter (*P*< 0.05). In the formula of adding 5 kinds of fish plasma and L15, the pH of Group LB was the highest, followed by Group LS, and the other 3 groups had little difference between 7.2 and 7.5. The osmotic concentration of Group LS was the highest, significantly higher than the other 4 groups (*P* < 0.01), but there was no significant difference among Group LC, LY and LB (*P*> 0.05).

**Table 4:** Measurement of pH and osmotic concentrations of different mix media in the beginning of culture time.

Conditions	L15 (ml)	M199 (ml)	Plasma		Mix antibiotic (ml)	pH	Osmotic concentrations (mOsm/Kg)
			Species	Volume (ml)			
L0	2			0	0.5	6.9∼7.2	0.261 ± 0.002^a^
M0		2		0	0.5	7.8∼8.0	0.250 ± 0.001^b^
LS	2		Skewband grunt	1	0.5	7.6∼7.7	0.285 ± 0.003^c^
LR	2		Red drum (M)	1	0.5	7.4∼7.6	0.278 ± 0.002^d^
LC	2		Common carp	1	0.5	7.2∼7.3	0.264 ± 0.002^a^
LY	2		Yellow catfish	1	0.5	7.3∼7.5	0.263±0.002^a^
LB	2		*B*.*capito*	1	0.5	7.8∼7.9	0.269 ± 0.002^a^

Osmotic concentrations labeled with different letters are significantly different (*P*<0.05).

### Comparison of fish plasma nutrients

#### Difference of main biochemical components in 5 kinds of fish plasma

**Fig 4:**
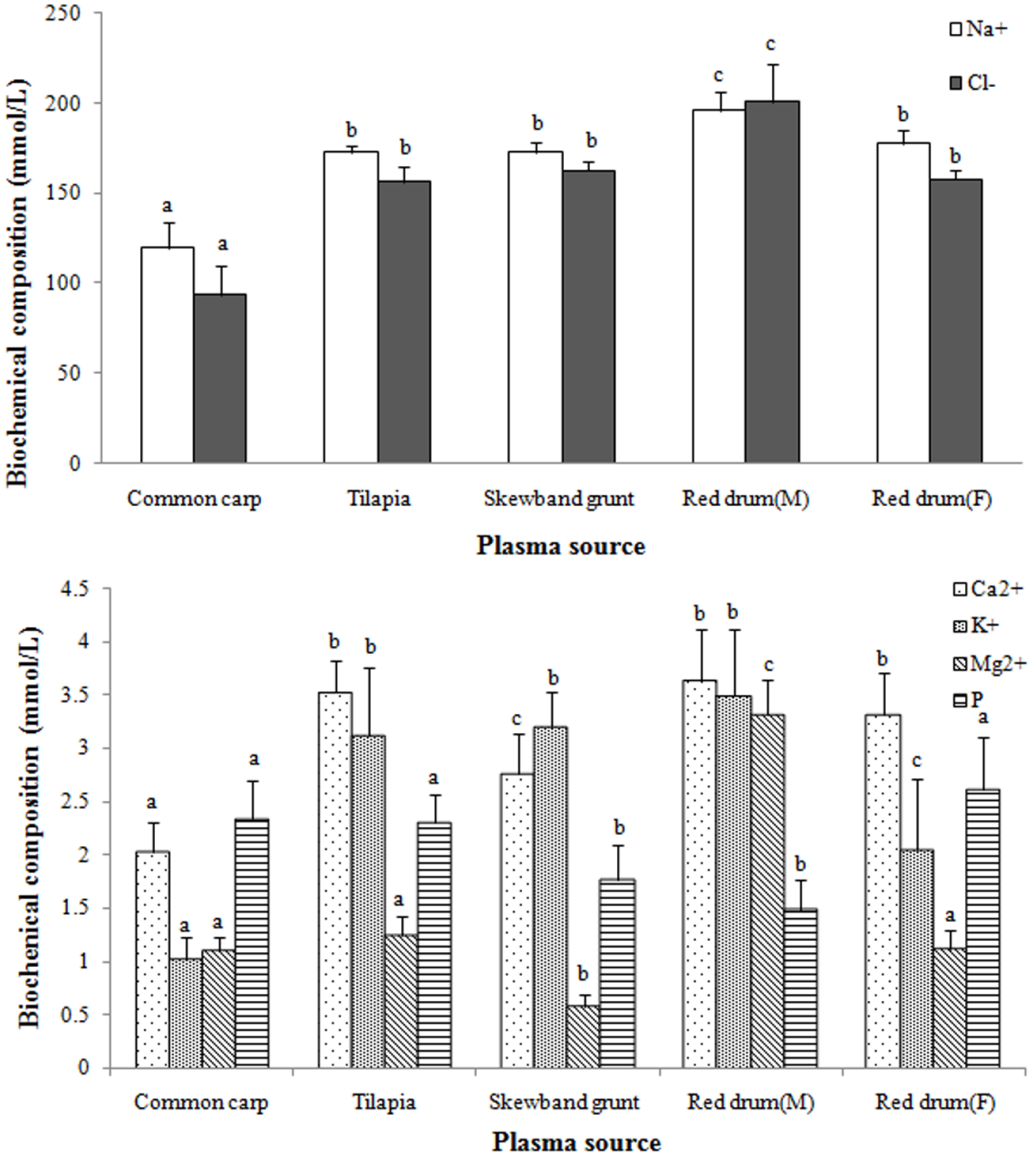
Comparison of 6 main electrolytes in 5 kinds of fish plasma. The same biochemical compositions labeled with different letters are significantly different (*P*< 0.05).

The difference of 6 main electrolytes of 5 fish plasmas was shown in Fig 4. The Ca^2+^ and Mg^2+^ content in skewband grunt were significantly lower than 2 kinds of red drum(*P* < 0.05), while there was no observed difference in the other 4 electrolytes when compared with one of red drum (*P* > 0.05). The plasma of skewband grunt had significantly higher Na^+^, Cl^-^, Ca^2+^and K^+^, and lower Mg^2+^ and P than common carp, but observed higher Mg^2+^, Ca^2+^, P and similar contents of Na^+^, Cl^-^ and K^+^ than tilapia. Compared with the marine group, the contents of Na^+^, Cl^-^, K^+^ and Mg^2+^ in desalinated red drum significantly decreased (*P* = 0.01), while P significantly increased (*P* < 0.01) with slight decline of Ca^2+^ (*P* = 0.123).

**Fig 5:**
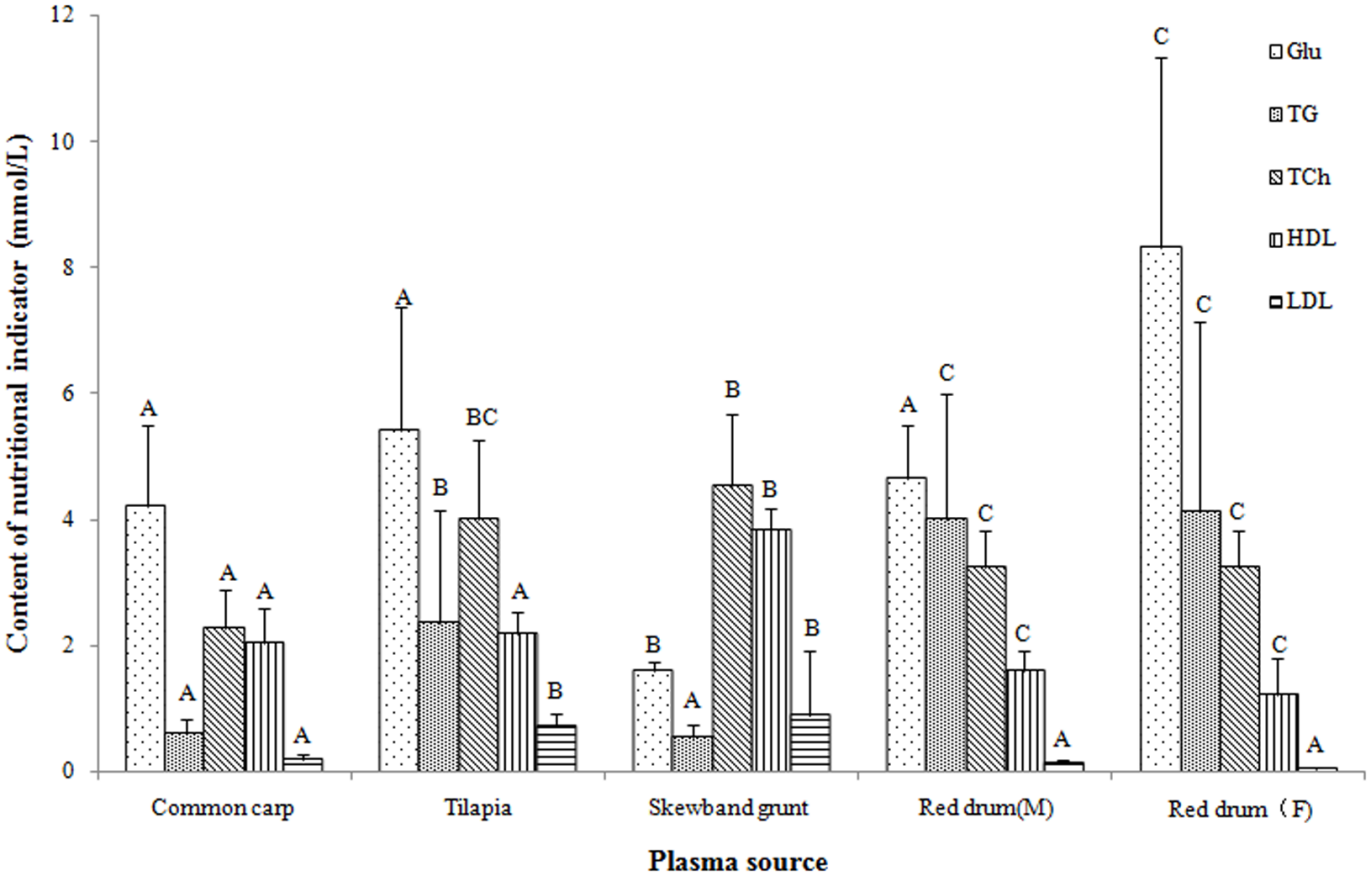
Comparison of main nutritional indicators in 5 kinds of fish plasma. The same biochemical compositions labeled with different letters are significantly different (*P*< 0.05).

The difference of main nutrition indictors was shown in Fig 5. The plasma of skewband grunt had significantly lower Glu (*P* < 0.05) and higher HDL (*P* < 0.01) than the other 4 fish species. TG of skewband grunt was significantly lower than 2 kinds of red drum (*P*< 0.01) and tilapia (*P* = 0.031) but common carp (*P* = 0.939); while TP was significantly higher than common carp, tilapia and marine red drum (*P*< 0.05) but desalinated red drum (*P* = 0.887); and LDL was significantly higher than common carp and 2 kinds of red drum (*P*< 0.001) except tilapia (*P* = 0.382). Compared with the marine group, the plasma of desalinated red drum had significantly higher TP (*P* = 0.01), Glu (*P* = 0.04) and LDL (*P* = 0.018), while there were no observed changes in TG, HDL and TCh (*P* > 0.05).

#### Difference of free amino acid in 4 kinds of fish plasma

Total of 22 free amino acids (FAA) were detected in 4 kinds of fish plasma, including 20 kinds of proteinaceous amino acid, and taurine, hydroxyproline (shown in S2 Table). The total FAA of common carp was significantly higher than skewband grunt, red drum and tilapia (*P*< 0.01). Comparing of single of FAA, there were 8 kinds in common carp significantly higher than 3 other fish species, including His, Gly, Thr, Ala, Val, Met, Leu and Tau(*P*< 0.05); and 8 kinds of FAA had no significant difference, including Asp, Glu, Ser, Tyr, Cyss, Phe, Asn and Hyp (*P* > 0.05). The plasma of common carp had significantly higher Arg and Trp than the skewband grunt, but there was no significant difference when compared with red drum or tilapia. Lys and Pro in skewband grunt were significantly higher than common carp, but there was no significant difference or significantly lower than red drum or tilapia; only Gln was significantly higher than the other 3 fish species.

#### Difference of fatty acid composition in 4 types of fish plasma

There were 21 kinds of fatty acid (FA) detected in 4 types of fish plasma, including 7 saturated fatty acids (SFA), 4 monounsaturated fatty acids (MUFA) and 10 polyunsaturated fatty acids (PUFA) showed in S3 Table. There were 14 kinds of FA in the plasma of skewband grunt significantly decreased (*P*< 0.01), and only C22:1 increased significantly (*P* = 0.04) when reserved for 11 months. The linoleic acid (C18:2), linolenic acid (C18:3), and C20:3 in common carp were significantly higher than newly prepared plasma of skewband grunt (*P*< 0.01), while the contents of C17:00, C20:2, C20:4 and C22:3 were not significantly different from those of at least one or more of the 3 other fish plasmas (*P*> 0.05). Combined with the difference of glochidial metamorphosis, there was only left 7 kinds of FA, including C15:0, C16:0, stearic acid (C18:0), oleic acid (C18:1), C22:0, DPA (C22:5) and DHA (C22:6), possibly related to the abnormal foot development of pink heelsplitter.

## Discussion

### Effects of culture methods on glochidial metamorphosis

L15 medium has different physiological salts comparing with M199 and MEM, and contains phosphate buffer system, which can maintain the culture medium pH in a certain physiological range in the condition of free CO_2_ [7]. We first compared the effect of L15 and M199 on metamorphosis of axe-head glochidia in pink heelsplitter, and the results showed that L15 medium benefit for the survival and metamorphosis of the glochidia at 24±0.5°C in the absence of CO_2_ supply. The osmotic concentration in the mixture with L15 was significantly higher than that of M199, and pH had greater variation. The gradient test indicated that there was no significant difference in glochidia metamorphosis rate when the osmotic concentration ranged from 245 to 290mOsm in vitro culture [7]. Therefore, it was concluded that the metamorphosis rate may be related to pH or nutrient composition of mixture culture medium.

We further selected out the optimal culture formula for pink heelsplitter from 14 kinds of plasma (serum) based on the improved method, which significantly simplified the culturing process and improved culture efficiency. It was the first success of non-parasitic metamorphosis of axe-head glochidium in vitro culture. The improved method has applied to in vitro culture of hooked, hookless and axe-head glochidia, and achieved satisfying results [21, 22]. It was proved that this technology had general applicability and especially important application value on artificial breeding and conservation of endangered freshwater mussel without host fish information.

### Effects of glochidia quality on metamorphosis

The investigation found that the glochidial metamorphosis rate in vitro culture was 0 when gravid mussel of *Lampsilis cardium* was reared in the inner artificial condition for 10 months. It was presumed that the quality of glochidia decreased gradually with the longer gravid time [7]. Our study showed that the metamorphosis rate in vitro culture can reach about 90% in the early reproduction season from Oct to Nov, but gradually and significantly decreased to about 30% in the late period from Mar to May of next year. It was completely consistent to the parasitic results of pink heelsplitter by Hua [18], and further confirmed Owen’s conclusion.

In nature, the pink heelsplitter spawn and fertilize eggs in Sep or Oct, house glochidia in the marsupium throughout the winter, but release them until to April or May. It is seemly not conducive to their natural reproduction, but may be the result of long-term co-evolution with natural host fish [23]. In captivity, the pink heelsplitter can reach sexual maturation and fertilization, but there was a great variation of number of glochidia among individuals with similar size, and asynchronous development even in the same marsupium. It was natural breeding characteristic, or affected by the lack of nutrition under artificial breeding or ecological condition, which needed further study. Generally, it was determined that the quality of glochidia was critical for its metamorphosis, but there was still no any criterion found to evaluate the quality.

### Morphological changes during the metamorphosis

In vitro culture, the procedure of glochidial metamorphosis was mainly divided into two stages. The first stage involved the glochidial adductor muscle degradation and mushroom body formation, and the second stage mainly involved internal tissue formation, including the digestive system, foot, gill, adductor muscle and so on [24,25]. Our study showed that glochidia not only completed the differentiation of internal organs, but the shell length increased to 2–3 times after metamorphosis. The external morphology of transformed juvenile changed from the original axe-head into nearly circular shape, which was consistent with morphology variation of juvenile mussel dropped from special host fish freshwater drum [18]. In process of glochidial metamorphosis of *H*. *bialatus*, the sensory hair, foot, adductor muscle and byssus gland of glochidia gradually degraded, simultaneously adductor muscle, foot, gills and digestive system of juvenile mussel gradually formed, whereas the glochidial shell and tiny ridges located on surface of abdominal margin was retained [26]. It was completely consistent with our observation in pink heelsplitter.

### Fatal factors in fish plasma to glochidia survival

Removing the serum complement in the media through heat inactivation also did not improve the survival or growth of the *L*. *fullerkati* juveniles produced in vitro culture [27]. Our results showed that the pink heelsplitter glochidia appeared rapid death in the media with addition of non heat-inactivated yellow catfish fish or *B*. *capito* plasma, while the glochidia in heat–inactivated groups had high survival rate and significant shell growth in 10^th^ day. It was concluded that there were some fatal factors existing in yellow catfish and *B*. *capito* plasma. Of 11 kinds of fish plasma added to the medium, the largemouth bass and hybrid sturgeon also showed internal tissue of glochidia rapidly decayed and died without shell growth in short time except yellow catfish and *B*. *capito*, which meant that these fatal factors might generally exist in some freshwater fish species. It should be one of important factors of high host specialists in pink heelsplitter, which also might be the first barrier of non-host fish to defend infection of glochidia of Unionidae.

### Potential nutrient limiting factors to glochidial metamorphosis

It was generally considered that the nutrition source of glochidia metamorphosis was mainly from the host fish blood and tissue, absorption of adductor muscle and degradation of the mushroom body [28,29], and the nutrient absorption pathway mainly depended on the phagocytosis or endocytosis of mushroom somatic cells transformed by mantle cells of glochidia [25,30]. Since Isom and Hudson firstly innovated in vitro culture method in 1982, all studies indicated that fish plasma or animal serum was essential for glochidia metamorphosis, but it remains unclear which nutritional factors in the plasma is critical for glochidia metamorphosis.

Of the 41 species that have achieved metamorphosis in vitro culture, 20 species can complete metamorphosis in the serum of rabbit or equine [9], but some species had observed different metamorphosis rate with addition of different plasma. The metamorphosis rate of glochidia of *H*. *myersiana* in vitro culture with addition of common carp and Nile tilapia plasma was significantly higher than striped catfish (32.42+5.85%) and equine serum (44.3+8.9%) [12]. The results indicated that freshwater mussel *Utterbackia imbecillis* could complete metamorphosis in bovine serum, rabbit and common carp sera in which glochidial metamorphosis rate were significantly higher than the two formers, and three freshwater mussels including *Alasmidonta viridis*, *Cyprogenia stegaria* and *Epioblasma capsaeformis*, failed to metamorphosis in rabbit serum, but in common carp plasma [7]. Ma *et al*. compared the metamorphosis rate of cockscomb mussel in L15 basic medium supplemented with plasma of one host fish or two non-host fishes respectively, but the results showed that the metamorphosis rate was significantly higher in two non host fish plasma than the host fish [22]. Owen [7] believed that some freshwater mussel species had obvious host specificity, which might require specific plasma or nutritional factors to complete glochidial metamorphosis and development, but not in mammalian serum due to absence of some essential nutrient factors. Our results showed that glochidia of pink heelsplitter fulfilled metamorphosis in vitro culture only with addition of plasmas of skewband grunt or red drum, which was consistent with its high host specificity and further confirmed the view of Owen [7]. It was concluded that there were critical nutrient factors for metamorphosis of pink heelsplitter existed in plasma of skewband grunt, but insufficient in red drum, and absent or in very low level in the other 12 plasmas (sera).

Comparison and analysis of the main biochemical indexes of 5 representative fishes indicated that the skewband grunt had marked lower blood sugar and triglyceride, higher protein, higher cholesterol and lipoprotein. Although major electrolytes and nutrition indicators of plasma appeared great variation between marine and designated red drum, there was no significant difference in the glochidia survival and metamorphosis rate. It was speculated that these indicators were not nutrient limiting factors of glochidial metamorphosis of pink heelsplitter.

Of note, the glochidial shell observed grew faster in the 20%, 50% and 100% common carp plasma groups than100% skewband grunt plasma, but the metamorphosis rate and vitality decreased significantly with the increase of common carp plasma. It was speculated that there were critical nutrient factors promoting glochidial shell growth in common carp plasma, but absent of nutrient factors promoting inner tissue development.

Most parasites had to rely on the uptake of FAA from the host or the amino acids produced from the degradation of the protein to meet their own needs [31]. Although 8 kinds of FAA in common carp plasma were significantly higher than the other 3 kinds of fish, their contents were very low and could be ignored when comparing to L15 added in mixture culture medium. The taurine of common carp plasma was 9.1 times of skewband grunt, 3.5 times of red drum and 3.6 times of tilapia. Due to no taurine in L15, it was assumed that high concentration of taurine was the only amino acid factor that may result in rapid shell growth in common carp, but it remained to be confirmed by further studies.

Parasites need lipids to maintain their survival in vitro. For example, serum is generally required in the culture of protozoa because cholesterol and some fatty acids in the serum are essential for their survival in vitro. However, the trypanosome endocytoses LDL of host by specific receptor locating in the flagellar pocket subsequently degrades the ApoB for utilization [31]. The contents of TG and LDL in the plasma of tilapia infested of glochidia of *H*. *cumingii* were significantly lower than blank group, but cholesterol increased slightly [32]. As a special parasite, glochidia of freshwater mussel are likely to utilize TG and LDL of the host fish as important lipid sources for their metamorphosis and development. Our results showed that LDL and TCh in skewband grunt plasma were the highest, and TG was the lowest among 5 kinds of representative fish. It was concluded that LDL and TCh may be a major lipid source of glochidial metamorphosis and development.

Combined with difference of glochidia metamorphosis in 11 kinds of fish plasma, it was concluded that there were 7 kinds of fatty acids possibly involving abnormal development of food and internal organs of juvenile mussel, and the correlation with polyunsaturated fatty acids DPA and DHA was especially significant.

The content of DHA in new collecting plasma of skewband grunt was 1.3 times of reserved for 11 months, 1.9 times of red drum and 10.6 times of common carp. DHA belongs to n-3 series of unsaturated fatty acids, which is the main component of phosphatide of brain and retinal nervous system, and plays an important role in the formation, growth, proliferation, differentiation of brain neurons, glial cells, and nerve conducting synapses. The deficiency of DHA could lead to fetal brain cell growth abnormalities and the central nervous system disorder during human embryo development from the third to sixth months [33]. At present, the function and requirement of DHA are still unknown in the stage of embryonic development and glochidial metamorphosis in Unionidae. Our study indicated that the vitro culture glochidia failed to transform in common carp plasma group, with abnormal foot only extending from anterior or posterior side, and its shell can’t open normally. The abnormal development in the skewband grunt plasma reserved for 11 months was similar to that of common carp plasma group. It was found there was significant native correlation between DHA content and abnormal rate of transformed juvenile. Therefore, the lack of DHA in mixed medium may be one of the important reasons of foot and visceral abnormalities. In addition, DHA can inhibit the synthesis of endogenous cholesterol and triglyceride, promote metabolism of TCh, TG and LDL, reduce blood lipids, and promote the elevation of HDL. The plasma of skewband grunt had significant lower TG but higher HDL than red drum and tilapia, which also might be related to its high content of DHA.

The content of DPA in skewband grunt plasma newly prepared was 1.9 times of reserved 11 months, 3.2 times of red drum and 9.6 times of common carp. As DHA, DPA also belongs to n-3 series of unsaturated fatty acids, which is one of the important intermediate products of synthesis of DHA, and has biological functions of protecting nerve and improving memory, anti-atherosclerosis, anti-inflammation and inhibiting platelet aggregation [34–36]. Because DPA content in skewband grunt plasma had similar difference as DHA comparing to other fishes, it was one of potential nutrient limiting factor for glochidial metamorphosis of pink heelsplitter.

### Mechanism of its high host specialists in pink heelsplitter

**Fig 6:**
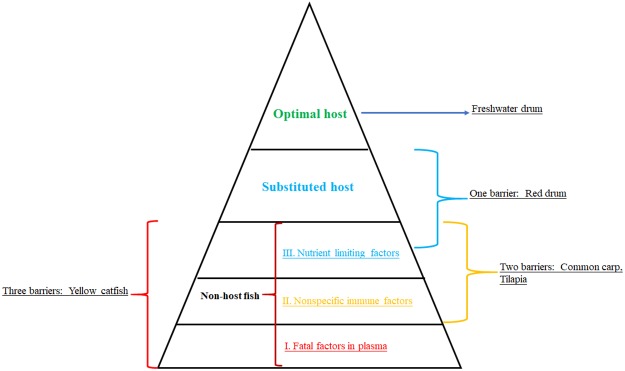
The mechanism of glochidia of host specialists in pink heelsplitter. There are three defensive barriers existing in non host fish, the first is fatal factors in the plasma, the secondary is nonspecific immune factors, and the third is nutrient limiting factors.

According to the results of host fish selection for pink heelsplitter, it was proved that freshwater drum was only optimal host fish, and red drum was potential host fish, while the other 14fishes, including yellow catfish, Nile tilapia, red tilapia, dark sleeper, crucian carp, globefish, yellow perch, Chinese perch, common carp, stone moroko, Wuchang bream, smallmouth bass, blue catfish, and brown bullhead catfish, were non host fish [20]. The results also showed that all glochidia infested in non host fish detached in 2–3 days. It was considered that nonspecific immunity of non host fish was main factor of glochidia selective infection or detachment prior to metamorphosis because the special antibody could be inspected at least 7 days after glochidia infection [37–39]. Combined with the results in present study, it seemed that there were three defensive barriers for host specialists in pink heelsplitter, which formed a 5 layer pyramid (shown in Fig 6). The first barrier was certain fatal factors existing in the plasma of small parts of non host fish, the secondary barrier was nonspecific immune factors exiting in most of non host fish, and the third barrier was nutrient limiting factors exiting in much more non host fish. Based on our mechanism of its host specialists in pink heelsplitter, yellow catfish had all three defensive barriers, Nile tilapia had two barriers, including nonspecific immune factors and nutrient limiting factors, and red drum was mainly affected by nutrient limiting factors and became substituted host. Only freshwater drum completely crossed all three defensive barriers and became optimal host fish of pink heelsplitter.

On the other hand, the yellow catfish was optimal host fish for the triangle pearl mussel but not cockscomb mussel, whereas the plasma was suitable for both glochidial metamorphosis in vitro culture [22,32]. It was concluded that the fatal factors to glochidia of pink heelsplitter in the plasma of yellow catfish was not lethal to triangle pearl mussel and cockscomb mussel, and the nutrient was sufficient for metamorphosis of these two species. Therefore, if this mechanism expands to all Unionidae species, only those fish passed through all three defensive barriers could become the optimal host fish. It means that there is no any one of three defensive barriers for glochidia of host generalists in most fishes, but exist any one, two or all of three barriers for glochidia of host specialists.

### Conclusion

It was proved that our improved method utilized L15 as basal medium significantly simplified the process of glochidial vitro culture and improved culture efficiency comparing to classical method. The improved method could be applied for vitro culture of hooked, hookless and axe-head glochidia. Based on this improved method, we firstly succeeded in non-parasitic metamorphosis of pink heelsplitter which had axe-head glochidia. Our results revealed that plasma of skewband grunt was the optimal nutrition for metamorphosis of glochidial in pink heelsplitter and DPA and DHA might be one of most important nutrient limited factors. We firstly found there were three barriers including fatal factors, nonspecific immune factors and nutrient limiting factors existing in non host fish, and tentatively established a pyramid mode for mechanism of selective parasitism and co-evolution between Unionidae and fish, which would provide important theory reference for reproduction and conservation of unionids of the world.

## Supporting information

S1 Table Analysis of free ammonia acid in 4 kinds of fish plasma. Different lowercase in the same line indicates significant difference (*P* < 0.05).
(DOCX)

S2 Table Component analysis of fatty acid in 4 different kinds of fish plasma. The contents are in mg/L. Different lowercase in the same line indicates significant difference (*P* < 0.05).
(DOCX)

S3 Table Information of fish selected for plasma collection. There is no English name for *Barbus capito*.
(DOCX)
