# Supplementary material for: Vitro culture of axe-head glochidia in pink heelsplitter Potamilus alatus and mechanism of its high host specialists
Source: PLoS One. 2018 Feb 15;13(2):e0192292. doi: 10.1371/journal.pone.0192292 (PMC5813935; doi:10.1371/journal.pone.0192292)
Supplement: S1 Table — Different lowercase in the same line indicates significant difference (P < 0.05). (DOCX) [file pone.0192292.s001.docx]

**S1 Table. Information of fish selected for plasma collection**

| English name | Scientific name | Toxonomy | Habitat | Range of body weight（g） |
| --- | --- | --- | --- | --- |
| Common carp | *Cyprinus carpio* | Cypriniformes, Cyprinidae | Freshwater | 100～200 |
| Nile tilapia | *Tilapia nilotica* (linnaeus) | Perciformes, Cichlidae | Freshwater | 400～600 |
| Yellow catfish | *Pelteobagrus fulvidraco* | Siluriformes, Bagridae | Freshwater | 100～150 |
| Yellow croaker | *Larimichthys crocea* | Perciformes, Sciaenidae | Marine water | 450～650 |
| Skewband grunt | *Hapalogenys nitens* (Richardson) | Perciformes, Pomadasyidae | Marine water | 450～600 |
| Marine bass | *Lateolabrax japonicus* | Perciformes, Moronidae | Marine water | 500～600 |
| Red drum(M) | *Sciaenops ocellatus* | Perciformes, Sciaenidae | Marine water | 500～750 |
| Red drum(F) | *Sciaenops ocellatus* | Perciformes, Sciaenidae | Salinity 5‰ | 500～750 |
| Largemouth bass | *Micropterus salmoides* | Perciformes, | Freshwater | 450～550 |
| / | *Barbus capito* | Cypriniformes, Cyprinidae | Freshwater | 500～600 |
| Hybrid sturgeon | *Acipenser baerii*(♀) *×A.schrenckii*(♂) | Acipenseriformes | Freshwater | 500～600 |
