# Supplementary material for: Vitro culture of axe-head glochidia in pink heelsplitter Potamilus alatus and mechanism of its high host specialists
Source: PLoS One. 2018 Feb 15;13(2):e0192292. doi: 10.1371/journal.pone.0192292 (PMC5813935; doi:10.1371/journal.pone.0192292)
Supplement: S2 Table — The contents are in mg/L. Different lowercase in the same line indicates significant difference (P < 0.05). (DOCX) [file pone.0192292.s002.docx]

**S2 Table. Analysis of free ammonia acid in 4 kinds of fish plasma**

| Amino acid | Common carp | Skewband grunt | Red drum | Tilapia |
| --- | --- | --- | --- | --- |
| Asp | 0.27±0.08^a^ | 0.33± 0.09^a^ | 0.39±0.00^a^ | 0.29±0.18^a^ |
| Glu | 0.18±0.13^a^ | 0.08±0.00^a^ | 0.08±0.01^a^ | 0.19±0.24^a^ |
| Ser | 0.06±0.02^a^ | 0.11±0.06^a^ | 0.14±0.06^a^ | 0.14±0.06^a^ |
| His | 0.25±0.11^a^ | 0.02±0.00^b^ | 0.07±0.00^b^ | 0.07±0.01^b^ |
| Gly | 1.25±0.19^a^ | 0.88±0.05^b^ | 0.56±0.13^c^ | 0.52±0.07^c^ |
| Thr | 0.45±0.07^a^ | 0.26±0.04^b^ | 0.19±0.09^b^ | 0.21±0.05^b^ |
| Arg | 0.07±0.00^a^ | 0.04±0.01^b^ | 0^c^ | 0.08±0.01^a^ |
| Ala | 0.43±0.09^a^ | 0.18±0.05^b^ | 0.26±0.04^b^ | 0.16±0.04^b^ |
| Tyr | 0.02±0.02^a^ | 0.01±0.00^a^ | 0.01±0.01^a^ | 0.01±0.00^a^ |
| Cyss | 0.01±0.00 | 0.01±0.00 | 0.01±0.00 | 0.01±0.00 |
| Val | 0.40±0.03^a^ | 0.29±0.05^b^ | 0.29±0.04^b^ | 0.23±0.03^b^ |
| Met | 0.07±0.01^a^ | 0.05±0.01^b^ | 0.05±0.01^b^ | 0.03±0.01^c^ |
| Phe | 0.12±0.01^a^ | 0.10±0.01^a^ | 0.11±0.02^a^ | 0.10±0.01^a^ |
| Ile | 0.17±0.01^a^ | 0.14±0.01^ab^ | 0.12±0.03^b^ | 0.07±0.01^c^ |
| Leu | 0.41±0.03^a^ | 0.26±0.04^b^ | 0.24±0.03^b^ | 0.17±0.02^c^ |
| Lys | 0.16±0.03^a^ | 0.32±0.06^b^ | 0.37±0.04^b^ | 0.11±0.05^a^ |
| Pro | 0.06±0.05^a^ | 0.27±0.07^b^ | 0.46±0.13^c^ | 0.11±0.05^a^ |
| Asn | 17.46±10.22^a^ | 26.68±11.72^a^ | 14.82±10.81^a^ | 23.90±9.40^a^ |
| Gln | 38.42±0.59^a^ | 58.15±11.11^b^ | 13.16±10.31^c^ | 8.09±2.27^c^ |
| Trp | 3.06±0.11^a^ | 2.10±0.33^b^ | 2.74±0.06^a^ | 4.50±0.41^c^ |
| Tau | 192.28±61.08^a^ | 21.16±2.67^b^ | 53.84±28.60^b^ | 55.34±12.47^b^ |
| Hyp | 58.13±3.84^a^ | 51.50±9.27^a^ | 49.32±17.47^a^ | 53.67±1.89^a^ |
| TAA | 313.7±63.30^a^ | 162.93±34.22^b^ | 137.22±44.61^b^ | 148.01±9.48^b^ |

Note: Different lower case in the same line indicates significant difference（*P* < 0.05）
