# Supplementary material for: Vitro culture of axe-head glochidia in pink heelsplitter Potamilus alatus and mechanism of its high host specialists
Source: PLoS One. 2018 Feb 15;13(2):e0192292. doi: 10.1371/journal.pone.0192292 (PMC5813935; doi:10.1371/journal.pone.0192292)
Supplement: S3 Table — There is no English name for Barbus capito. (DOCX) [file pone.0192292.s003.docx]

**S3 Table. Component analysis of fatty acid in 4 different kinds of fish plasma**

| Fatty acid | Skewband grunt  (New) | Skewband grunt  (Reserved 11M) | Red drum | Common carp |
| --- | --- | --- | --- | --- |
| C14：0 | 101.5±5.7^a^ | 93.3±9.2^a^ | 45.7±2.0^b^ | 8.0±0.7^c^ |
| C15：0 | 36.5±1.6^a^ | 20.3±1.3^b^ | 13.8±1.2^c^ | 7.4±1.1^d^ |
| C16：0 | 2160.8±91.4^a^ | 1721.1±158.6^b^ | 1046.3±45.3^c^ | 384.1±20.3^d^ |
| C16：1 | 342.4±19.0^ac^ | 403.3±71.7^a^ | 311.9±2.7^c^ | 43.4±6.7^b^ |
| C17：0 | 71.2±1.0^a^ | 30.4±2.2^b^ | 18.4±2.5c | 20.2±4.3^c^ |
| C18：0 | 760.9±24.1^a^ | 490.3±45.5^b^ | 172.2±5.6^c^ | 72.6±8.8^d^ |
| C18：1 | 1465.1±62.8^a^ | 1224.6±164.9^b^ | 704.7±20.2^c^ | 189.1±10.4^d^ |
| C18:2 | 58.2±1.1^a^ | 92.2±10.8^b^ | 20.2±1.6^c^ | 130.4±5.5^d^ |
| C18:3 | 15.0±0.4^a^ | 22.4±0.8^b^ | 11.6±1.7^a^ | 21.7±4.2^b^ |
| C20:0 | 13.3±0.7^a^ | 12.6±1.2^a^ | 6.2±0.4^b^ | 2.0±0.2^c^ |
| C20:1 | 68.5±4.4^a^ | 67.0±13.0^a^ | 28.4±1.8^b^ | 12.4±0.5^c^ |
| C20:2 | 15.9±0.7^a^ | 10.9±0.7^b^ | 4.4±0.4^c^ | 11.1±0.5^b^ |
| C20:3 | 13.7±0.4^a^ | 6.2±0.7^b^ | 2.5±0.3^c^ | 23.6±1.9^d^ |
| C20:4 | 341.1±8.5^a^ | 161.6±6.8^b^ | 138.1±11.2^c^ | 128.2±11.2^c^ |
| C20:5 (EPA) | 375.0±15.9^a^ | 419.3±48.3^a^ | 315.6±0.5^b^ | 105.2±28.8^c^ |
| C22:0 | 9.3±0.4^a^ | 5.4±0.7^b^ | 3.4±0.4^c^ | 2.0±0.3^d^ |
| C22:1 | 19.0±0.7^a^ | 33.1±8.4^b^ | 10.5±0.3^c^ | 1.5±0.7^d^ |
| C22:3 | 129.3±26.5^a^ | 22.3±7.8^b^ | 20.8±3.1^b^ | 25.6±11.0^b^ |
| C22:4 | 65.3±2.9^a^ | 53.1±2.8^ab^ | 45.8±12.3^b^ | 30.9±4.8^c^ |
| C22:5 (DPA) | 335.0±14.9^a^ | 178.2±4.8^b^ | 104.6±6.2^c^ | 34.8±11.2^d^ |
| C22:6 (DHA) | 2406.9±63.1^a^ | 1833.9±187.1^b^ | 1272.1±88.7^c^ | 227.9±20.4^d^ |
| PUFA | 3755.6±123.6^a^ | 2800.1±235.6^b^ | 11935.8±114.2^c^ | 739.4±92.2^d^ |
| UFA | 5650.6±208.1^a^ | 4528.1±472.9^b^ | 2991.3±121.3^c^ | 985.9±109.0^d^ |

Note: Different lower case in the same line indicates significant difference（P < 0.05）. The contents are in mg/L.
